# PhysioDroid: Combining Wearable Health Sensors and Mobile Devices for a Ubiquitous, Continuous, and Personal Monitoring

**DOI:** 10.1155/2014/490824

**Published:** 2014-09-10

**Authors:** Oresti Banos, Claudia Villalonga, Miguel Damas, Peter Gloesekoetter, Hector Pomares, Ignacio Rojas

**Affiliations:** ^1^Department of Computer Architecture and Computer Technology, Research Center for Information and Communications Technologies, University of Granada (CITIC-UGR), C/Periodista Rafael Gomez Montero 2, 18014 Granada, Spain; ^2^CGI Spain, Avenida de Manoteras 32, 28050 Madrid, Spain; ^3^Department of Electrical Engineering and Computer Sciences, Muenster University of Applied Sciences, Stegerwaldstraße 39, 48565 Steinfurt, Germany

## Abstract

Technological advances on the development of mobile devices, medical sensors, and wireless communication systems support a new generation of unobtrusive, portable, and ubiquitous health monitoring systems for continuous patient assessment and more personalized health care. There exist a growing number of mobile apps in the health domain; however, little contribution has been specifically provided, so far, to operate this kind of apps with wearable physiological sensors. The PhysioDroid, presented in this paper, provides a personalized means to remotely monitor and evaluate users' conditions. The PhysioDroid system provides ubiquitous and continuous vital signs analysis, such as electrocardiogram, heart rate, respiration rate, skin temperature, and body motion, intended to help empower patients and improve clinical understanding. The PhysioDroid is composed of a wearable monitoring device and an Android app providing gathering, storage, and processing features for the physiological sensor data. The versatility of the developed app allows its use for both average users and specialists, and the reduced cost of the PhysioDroid puts it at the reach of most people. Two exemplary use cases for health assessment and sports training are presented to illustrate the capabilities of the PhysioDroid. Next technical steps include generalization to other mobile platforms and health monitoring devices.

## 1. Introduction

Healthcare is a pending matter that challenges worldwide [1]. The severe socioeconomic situation suffered by an important part of developed countries is providing greater pressure to find more cost-effective solutions to the provision of health and social care. Cuts in government spending, an increasing population of pensioners and a growing unemployment rate, are critical factors that add urgency to the need of finding new healthcare solutions. Moreover, healthcare systems in developing countries confront serious difficulties in providing care and assistance, mainly due to scarcity of personnel and resources. Information and communications technologies appear in this context to revolutionize this field and to provide innovative, efficient, and affordable solutions. In fact, a strong effort is being put by companies, research institutions, health organizations, governments, and other entities all over the world into promoting, showcasing, and catalyzing the use of new technologies in healthcare. Current trends demonstrate that the combination of the latest clinical knowledge with the cutting-edge technology paves the path to a new dimension of health and social care.

During the last years, several concepts have emerged as part of the new healthcare era. Medicine 2.0, Health 2.0/3.0, ePatient, and eDoctor, among other more established terms such as eHealth, telehealth, or telemedicine, are widely disseminated examples of these concepts. Most of the ideas behind these novel domains are devoted to increase patients' self-management, procure preventative care, and enhance health professional expertise. Particularly fundamental to this renovated healthcare paradigm is to make patients more participatory of their care process. To that end, patients should be equipped, empowered, enabled, engaged, emancipated, equals, and experts [2]. As to other areas, the Internet has been recognized to be the perfect medium to support these essential capabilities. For example, an enhanced access to health-related information on the web via semantic and networked resources could facilitate an improved understanding of common health issues [3]. Likewise, social networking [4], social media [5], and virtual reasoning [6] are meant to be primal enablers of the new health generation. Personalized social networks may foster the definition of supportive virtual communities, within which individuals can help one another understanding and managing different kinds of health-related issues. Moreover, these networks may serve as a means for health professionals to facilitate access to medical knowledge and improve timely communication with patients, of particular interest to increase acceptance and adherence to therapeutic treatments. Interaction through social media stands out as a more “human approach” to the individual seek of online health information. This idea takes advantage of the collectivity to support patients and family caregivers in their feelings of loneliness, reassure them in their behavior and daily efforts, and validate adopted medications, devices, and health services.

Although these innovative tools are truly interesting to promote a more personalized and independent healthcare, a crucial aspect that may not be approached through web technologies is corporeal monitoring. Body monitoring deepens into the patients' physiology, biological conditions, and behavioral aspects, which are utterly necessary to have a precise understanding of their status and particular necessities. According to the traditional health care model, patients' monitoring is normally relegated to sporadic doctor visits or institutionalization, which goes against the principles of proactivity, independence, accessibility, and cost-effectiveness. Oppositely, embracing these principles arises mobile Health (mHealth). mHealth is an emerging and rapidly developing field that builds on a wide range of mobile technologies such as smartphones, tablets, and portable health devices to support community and clinical health data retrieval, delivery of healthcare information, or direct provision of care. Most interestingly, mHealth covers technological solutions for the monitoring of patients' behavior and vital signs. The potential of mHealth stems from the capacity of making technology portable or even wearable [7]. Accordingly, systems may be used in an ubiquitous manner and provide seamless monitoring capabilities. As an example, remote health monitors may continuously inform caregivers or practitioners to respond fast in the event of an emergency [8] or a change in the patients' conditions [9]. Furthermore, not only could these devices be useful to enhance medical tasks but also to make them possible. These systems may become much valuable in regions where the trip to a care center takes several hours or a few doctors must assist thousands of patients. In this regard, mHealth emerges as a means to provide greater access to healthcare services to a broader segment of population. The evolution of electronics, getting smaller and cheaper, is supporting the access to more affordable solutions that satisfactorily overcome traditional communication barriers stemming from old-fashioned technologies. It is important to notice that even when mHealth technologies may not be in principle expected to be defrayable by low-income nations, reality shows that there exists a rapid rise of penetration in these countries. In fact, more than half of worldwide new smartphone users belong to developing countries, owing to the expansion of low-end smartphones, with the fastest growth on the Asian continent [10].

From a technological perspective, one of the main limitations of up-to-date portable health monitoring devices are processing and storage capabilities. Monitoring devices normally consist of a set of sensors that measure physiological magnitudes and convert them into machine readable information. Although some of these devices include dedicated resources to process the information, these are very sparse and limited. Conversely, mobile devices are the perfect means to collect the data retrieved from health monitoring devices, as well as to provide local processing capabilities or even grant access to cloud functionalities (high performance computation, large capacity storage, and data analytics) [11]. Through this, not only the data collected from the patient could be consider for their health assessment and care, but also the records from hundreds, thousands or even millions of patients, that may potentially share similar necessities and conditions. This massive collection and processing of health data is seen to greatly boost the medical understanding, thus leading to an optimal provision of care.

In this work, we present the PhysioDroid system, which combines both commercial wearable health devices and mobile devices to provide continuous and personalized patient physiological monitoring. An application that runs on the Android platform is implemented to enable the collection, sharing, and exchange of physiological data registered through the ubiquitous wireless health monitor. Other features such as health data visualization, storage, and alerts on conditions are further available from this. Firstly introduced in [12], the PhysioDroid system is part of the AdaBIO (Advanced Intelligent Systems for Biomedical and Bioinformatics Applications) project and also contributes to the WHM (Wireless Health Monitoring) and OPENi (Open-Source, Web-Based, Framework for Integrating Applications with Social Media Services and Personal Cloudlets) projects. In these projects adaptive methodologies, expert models, and ubiquitous interfaces are combined with noninvasive wireless sensors for multimodal biomedical monitoring. Moreover, it is aim of these projects to provide more robust wireless systems, improve healthcare monitoring, and leverage multiple user physiological data to principally lead to an earlier discovery, track of evolution, and trends prediction on patient conditions.

The rest of the paper is organized as follows.  Section 2 includes a brief summary of the latest ubiquitous health monitoring systems and forefront medical apps. The PhysioDroid system is introduced in Section 3, while their constituent elements are fully described in Sections 4–6.  Section 7 presents a couple of use cases where the PhysioDroid system is devised to potentially be exploited. Finally, main conclusions and future steps are presented in the last section of this paper.

## 2. State of the Art

Looking at the market for state-of-the-art mobile health care sensors, an estimated number of 16 million devices will be sold by the year 2016, as predicted by ABI Research [13]. These devices, which have been extensively used during the recent years in research for diverse appliances such as disease tracking [14, 15] or analysis of human behavior [16–18], are becoming more popular among end-users. The choice of which device to use for certain applications boils down to the features and incorporated sensors. Similarly, the way these devices are worn is very different just as the way they obtain data from the body or communicate with base stations for further diagnosis and presentation. Some of the manufacturers already provide software to examine the collected data on a handheld device or desktop computer, whilst others provide supervision of specialists from remote servers. An extensive updated list of more than 240 different mobile health devices is provided in [19].

Examples of specific purpose commercial health monitoring devices are the* Irythm Zio Patch* [20], a long-term cardiac rhythm monitor specially designed to improve prognosis of cardiac arrhythmias, the* fitbit* [21], for intake and activity assessment or the* Zeo Sleep Monitor* [22], which is specially indicated for sleep disorders analysis. The achievement of full hospital-grade is now also possible with the release of completely portable devices such as the* Smartheart* [23], the first personal mobile 12 lead ECG for the detection of ischemic cardiac events. Examples of more general commercial health monitoring devices are the* ViSiMobile* [24], which measures ECG, HR, oxygen saturation, BR, noninvasive blood pressure, and skin temperature; the* AVIVO MPM PiiX* [25], which is capable of monitoring fluid status, HR, BR, posture, activity, and ECG; the* Equivital LifeMonitor* [26], that collects ECG, BR, skin temperature, and acceleration data along with other metrics such as galvanic skin response, oxygen saturation, or geopositioning; the* Zephyr Bioharness* [27], a garment that registers comprehensive physiological data from medical-grade ECG, HR, and BR to motion; or the* RS TechMedic DynaVision* [28], which incorporates ECG, HR, plethysmogram, SpO2, and body temperature sensors.

The use of mobile medical applications has increased over and over during the last years. Apple's iOS platform has been demonstrated to be much more mature in this field; however, its use is tailored to a reduced and expensive catalog of devices. As an alternative, Android provides its users with a wider variety of systems of different prices and vendors at the reach of a broader audience [29, 30]. This reduced cost translates into a higher number of potential users from which the diverse stakeholders, including patients, relatives, caregivers, practitioners, institutions, and companies may benefit.

The vast majority of medical apps have informative and academic purposes, which make them specially recommended to professionals and students.* Medscape*,* Epocrates,* or* Eponyms* are popular examples of this category, particularly intended to provide comprehensive and updated information for medical procedures, disease monographs, drug references, or practice guidelines [31]. Other applications are most useful for primary care practitioners or generalists, such as* Calculate by QxMD*, which provides them with medical calculators and decision support tools that apply to General Practice, Internal Medicine, Cardiology, Surgery, Obstetrics, or Neurology, among others. Electronic reference manuals are also close at hand, as is the case of* Monthly Prescribing Reference (MPR)*, an app that incorporates prescribing notes and drug records to facilitate clinical practice and promote the access to the latest advances in treatments [32]. Health care applications are not just aimed at specialists, but also at other sort of users. This is the case of* DoctorMole*, which offers a first diagnosis of the malignancy of a given mole. To do so, the app makes use of the smartphone's built-in camera to take a picture from the affected area and superimposes the diagnosis directly on the picture through augmented reality. Another application that takes advantage of the camera to diagnose is* uCheck*.* uCheck* analyzes the color of the chemical strips dipped in a sample of urine. This is subsequently compared to a color-coded map and within a few seconds, a full report containing levels of glucose, bilirubin, proteins, ketones, or leukocytes is available to the user. Normally, the information is presented in an easy to understand format, which applies to both medical and general users interests. This type of tools could be of worth to identify potential diseases and track the evolution of detected illnesses. In turn, this may translate into more precise and proactive monitoring than usual quarterly/annual medical examinations.

Although there is a relevant number of medical apps that apply to diverse health domains, there are very few that support physiological monitoring. Examples of these scarce apps are* Instant Heart Rate* or* Cardiograph*, which use the smartphone's camera to get an accurate heart rhythm reading from the user fingertip. Mainly intended for entertainment purposes,* iStethoscope* also turns the mobile phone into a stethoscope, allowing the users to listen to their heartbeats and visualize the heart waveform on the screen. One of the major advantages of these apps, with respect to others, is the fact of not requiring additional devices to the own smartphone. However, they demonstrate inaccurate as well as cumbersome for continuous monitoring. Moreover, there exists almost no regulation for these apps, thus, making their public embracement and use difficult, despite the high interest shown by physicians and trainees [33, 34]. The use of more precise, professional, and validated mobile health care sensors, in conjunction with mobile devices, is then of mandatory consideration. Although this area has been little explored yet, some preliminary products such as* iBGStar*, an electronic blood glucose meter for diabetes management, or* iSpO2*, which measures the oxygen saturation level in the blood and heart rate, let us envision a promising future for mobile and ubiquitous health care.

## 3. PhysioDroid

PhysioDroid is an advanced ubiquitous system for remote and continuous monitoring of people's physiological and behavioral status. The PhysioDroid system builds on the combination of wearable health sensors, capable of measuring physiological and behavioral data, and mobile devices, in charge of gathering and processing the collected information. The system is particularly conceived to empower users in their daily living, as well as to make them conscious and participatory of their healthcare and well-being, through the access to a simplified description of their health status. PhysioDroid also provides track and alerts on conditions, as well as mechanisms to trigger emergency procedures at the point of need. Besides, the PhysioDroid system does not only apply to individual users, but it is devised to leverage health data collected from multiple users. This is seen to be a key accelerator of medical and social knowledge, helping carers to provide more efficient medical diagnoses, treatments and proactive policies.

**Figure 1:**
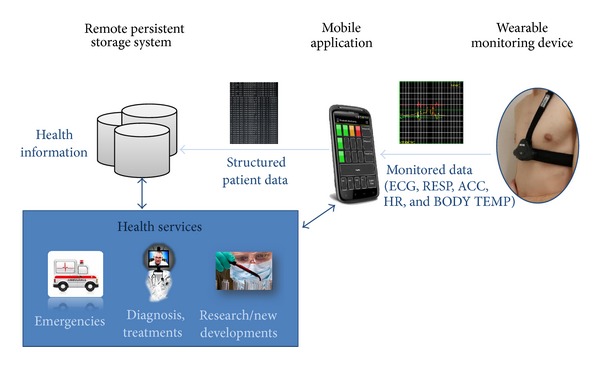
The PhysioDroid system. From right to left, the wearable monitoring device records the user's vital signs and transmits them to the mobile device. The application running on the mobile device provides functionalities such as visualization and interpretation of the collected data, triggering of alarms and health alerts. The mobile device also serves as gateway to the remote persistent storage system, which provides advanced health services based on the analysis of medical data from multiple users.

The PhysioDroid system consists of (Figure 1):

- a wearable monitoring device that records different types of physiological data on a subject and transmits them wirelessly;
- a mobile device, for example, smartphone, which runs an app that acts as collector of the data delivered by the vital sensor, support system for medical diagnosis and health alerts, interface for user data inspection and gateway to forward the data to a remote storage for further analysis;
- a remote persistent storage system to store data from multiple users, particularly devised to support advanced health services and analytics.

## 4. Wearable Monitoring Device

**Figure 2:**
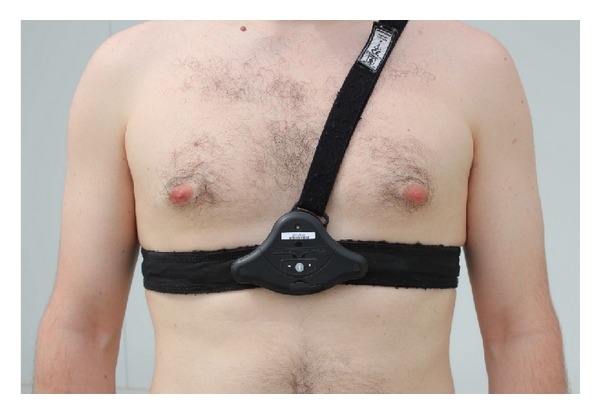
Equivital LifeMonitor device. It comprises the sensor electronics module (SEM) and the monitoring belt. The SEM contains a battery, electronics, and software, in order to process the measurements from the monitoring belt. The monitoring belt holds the SEM onto the body and contains fabric electrodes, which require contact with the user's skin to measure the body's vital signs.

The monitoring of the user's vital signs is performed through the Equivital EQ01 system (see Figure 2). The EQ01 [35] is a multiparametric, wireless, and portable health sensor device, that collects and transmits vital sign information measured from the body of the wearer to a smart computer, server or base station. Through a Bluetooth connection, the information may be sent over a network in close proximity to the device. The EQ01 is attached to the body with the help of a belt, which is strapped around the chest. Embedded on the belt and the back of the device are sensors that rest directly on the skin, to measure vital information through impedance measurements. The EQ01 senses electrocardiogram (ECG, 2-leads at 256 Hz, from which is derived the heart rate, HR), respiration (RESP, at 25.6 Hz through an embedded strain gauge), motion (acceleration, ACC, at 25.6 Hz), and body skin temperature (BODY TEMP). The device provides a very limited proprietary processing of the information, including filtering and low level knowledge extraction. Thus, for example, the device is able to detect a reduced set of postures (e.g., standing, lying) from the analysis of the measured acceleration or extract the R-R interval for electrocardiogram analysis. The sensor operation has been performance tested to the American National Standard (ANSI/AAMI EC13:2002). The performance results for this tests, as well as the models implementation are disclosed in [36]. Moreover, the EQ01 provides system status information as part of its main features. There are two operating modes available for the EQ01, full disclosure (which includes the raw data delivery) and partial disclosure (only calibrated data is provided). Here the first mode is considered to keep all the information registered by the system. The operational time of the device is superior to 24 h, although the vendor recommends not to wear the band more than a day at one time. For more specific details regarding communication, storage protocols, and information encoding, the reader is referred to the product manuals and datasheets [36].

## 5. PhysioDroid Mobile App

The data measured through the EQ01 is further transmitted to a mobile device (e.g., smartphone or tablet). In particular, Android devices have been here considered. Several advantages of the Android operating system with respect to its competitors were conclusive during the platform selection. These include the greatest growing mobile market, opensource framework, highest performance stability and security, and continuous updates and upgrades of the application programming interface (API), among others. Different mobile devices have been tested during development and validation of the system, including Samsung, HTC, LG, or Sony devices for a representative selection of the various Android API available versions (“Gingerbread” API 2.3 and newer). The average battery lasting time for most of the evaluated mobile devices, during full operation of the PhysioDroid system, is above ten hours. In the following, the PhysioDroid app is described.

### 5.1. App Usage

**Figure 3:**
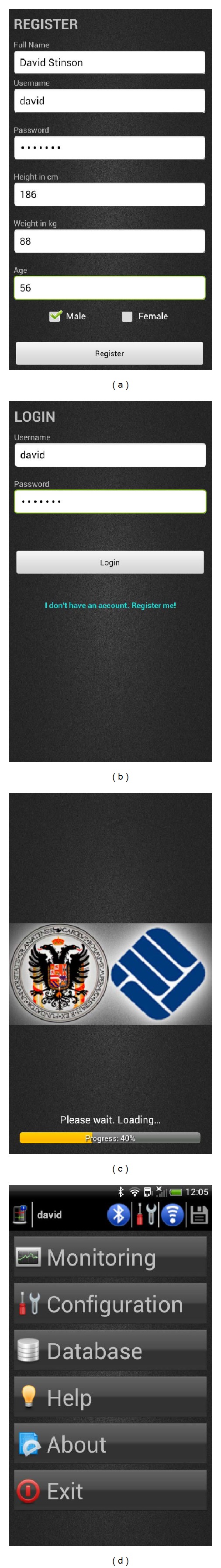
Screenshot from the application wizard to get started. (a) Registration. (b) Login. (c) Modules loading. (d) Main menu.

The process to get started on the app is depicted in Figure 3. After launching the app, the user is asked to insert name and password, to uniquely identify and log into the application contents (Figure 3(b)). Registration is only required for the first time access (Figure 3(a)). Here, the user is asked to insert some data related to their personal information and health profile, such as name, age, weight, height, and gender. This information is then available for some of the planned recommendations and alerts. Thereupon the user is logged and the application modules loaded (Figure 3(c)), the main menu is available (Figure 3(d)). The principal items of this menu are “Monitoring,” “Database,” “Configuration,” and “Help.”

By pushing the button “Monitoring,” the user starts the monitoring procedure and gets access to some of its main functionalities. First, the app triggers the process of binding both mobile device and EQ01. To that end, the user is asked to allow the Bluetooth connection. As soon as both mobile device and EQ01 are bound, the application starts collecting the data delivered by the wireless monitoring device. The EQ01 raw data are encoded into the PhysioDroid proprietary format to be used by other application functionalities. These application functionalities principally enable data processing and real-time visualization on the mobile device.

**Figure 4:**
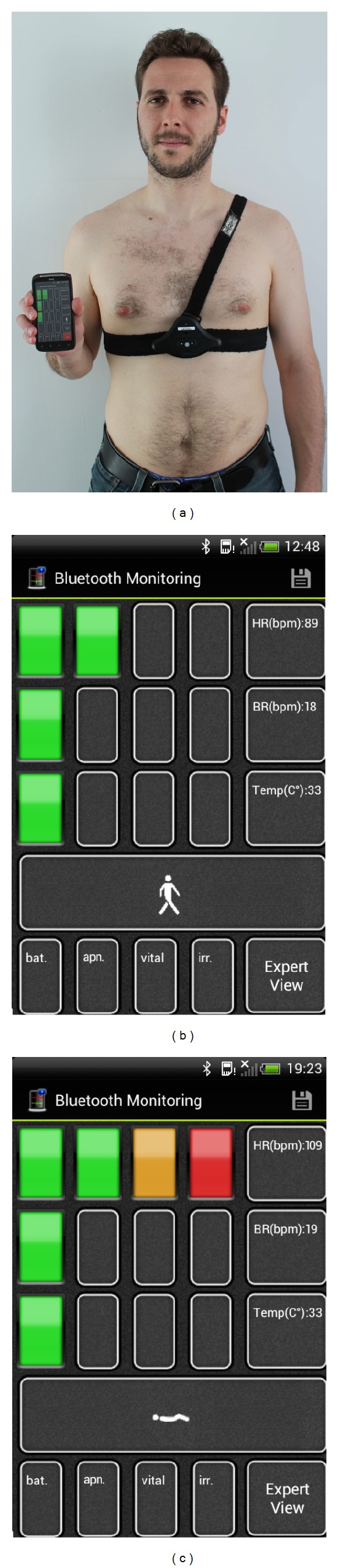
(a) User wearing the EQ01 monitor while holding a smartphone running the PhysioDroid app. (b) Screenshot from the user monitoring view. Example for an apparently normal physiological status. (c) An anomalous situation regarding the measured HR is alerted.

Two visualization modes are provided, one for the experienced analyst and other for the average user (see Section 7). The* expert view* provides real-time representation of physiological data waveforms. This is particularly suited for clinical specialists that may require a precise description of the recorded information. Amongst others, the ECG, acceleration, and respiration waveforms may be displayed, as well as the skin temperature. A simpler presentation of the health data is provided for the regular users. The* user view* includes averaged values (HR, BR, and TEMP) and visual indicators that inform about the status of the monitored vital data (Figures 4(b)-4(c)). For a user-friendlier inspection of the vital signs several status bars are defined. Each bar refers to a numerical range that identifies the adequacy of the measured physiological parameter. To do so, a number of bars light up from left to right and green to red (green = “OK,” yellow = “warning,” and red = “danger”). From here, the user may check and react to data, for example, by following personalized clinical guidelines or recommendations. The thresholding of these states (bars) is devised to be set by a medical expert depending on the particular health conditions of the user. In fact, the app has been defined in a way that users only need to provide the URL of their health care provider, through the “Configuration” menu. Therefore, the app automatically connects to the health care provider service, which provides the threshold values to be set in the app for the particular patient characteristics. By default, and for evaluation purposes, the app natively provides a set of predefined threshold ranges, which nevertheless do not strictly generalize to all people and conditions.

As a key feature, it has been implemented the possibility of triggering an emergency call when a specific event occurs (e.g., when a threshold is largely exceed). The phone number to whom the calls should be addressed could be also set through the “Configuration” menu. The emergency call is directed to the “112” by default, in order to reach emergency services. Given the costs of triggering a false alarm, and although the EQ01 sensor holds various medical certifications that supports its reliability, specific situations that may lead to an erroneous alert have been especially considered. This refers, for example, to the case when the user removes the vital band before ending the monitoring process or the communication between mobile and wearable devices is lost.

For the motion data interpretation, a stick that imitates the identified posture or exercise is particularly used. At the bottom of the screen the user also gets information about the EQ01 battery status, apnea occurrence, vital signs, and irregular heart beat events. All these events come from the data provided by the EQ01.

**Figure 5:**
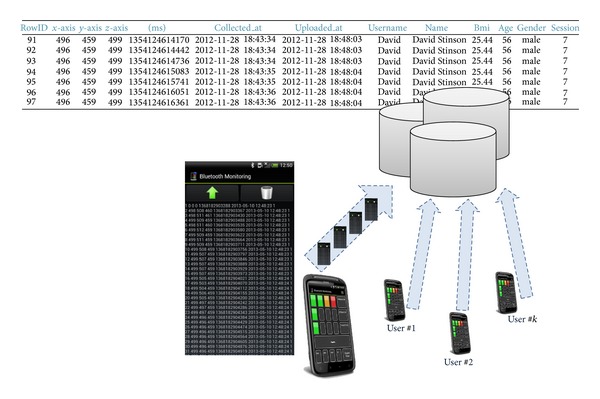
The PhysioDroid app may be used as a gateway to upload the monitored data to a remote persistent storage unit. This action may be performed either manually or by setting up a routine for the upload process.

The user may display some of the stored data through the “Database” menu (see Figure 5). Nevertheless, this has been rather defined for those users that wish to manually upload the data to the remote persistent storage system or to remove them whether necessary. By default, the PhysioDroid app uploads the data automatically; however, this may be changed through the configuration options. These kind of functionalities are better thought for the sake of research. Storage procedures are described in Section 6.

Apart from the functionalities already explained for the “Configuration” menu, here the user may also change other application settings such as data uploading interfaces (WIFI/3G) or user profile updates. All functionalities and the* HowTo* are neatly described in the manuals that can be access through the “Help” menu.

### 5.2. App Implementation

The PhysioDroid app consists of seven packages named according to the functionality they provide:* cache*,* drawing*,* login*,* monitoring*,* upload*,* storage,* and* add-ons*.

The* cache* package prototypes classes that implement a cache memory based on a buffer. This allows the application to perform on-the-fly data processing without requiring local permanent storage (e.g., microSD card). In fact, the use of a persistent storage is observed to slow down significantly the processing of the data or at worst prevent it.

The incoming data is managed and administered by the* monitoring* package, which further supports the Bluetooth connections between the vital monitor and the mobile device. These connections are established through the Bluetooth radio frequency communication protocol (RFCOMM, [37]) and the serial port profile (SPP, [38]), which emulates a serial port connection.

The* storage* package manages local persistence processes. The storage functionalities build on SQLite [39], which is a popular database engine on memory constrained systems, like mobile devices, since it runs in minimal stack space and very little heap. SQLite defines a compact in-process library that implements a stripped version of SQL.

The* upload* package controls remote storage functionalities. The transmission to the remote storage is implemented using a HTTP POST request method, which encloses in the message's body the SSL-encrypted representation of the data.

Graphs and general data presentation are implemented through the* drawing* package. This package implements some of the native functions provided by the Android API.

User login and registration are handled within the* login* package. Here again, Android native functionalities are used to implement authentication and authorization mechanisms. Finally, the* add-ons* package contains classes that are not essentially needed for the application core functionalities but provide features to make the user experience more pleasant (e.g., manuals, guidelines).

The implemented software is built on Android API levels 9 to 15 and makes use of the best practices recommended by Google in order to improve user experience, reduce power consumption, and optimize performance. The software modularity also allows developers to easily include new functionalities for signal acquisition, data storage, data analysis, and data transmission as well as other add-ons whether required. In fact, the PhysioDroid app has not been defined as a closed application but as a primary tool that may be specifically configured to approach different medical and health-related problems.

## 6. Remote Persistent Storage System

The third component of PhysioDroid is the remote persistent storage system. Physiological data collected by the wearable monitoring device may be transmitted through the mobile app to a remote storage component. The app can be operated without making use of this remote component; however, in this case the spectrum of value-added services would be more limited.

Remotely-stored health data could be accessed by doctors in order to control the patients' vital information or by any other authorized third party, for example, a clinician. Moreover, the collected data could be further processed to infer some knowledge about the users' physiological conditions, or even to determine behavioral patterns of a certain demographic group, for example, elderly.

The current implementation of PhysioDroid uses a remote server to provide the storage capabilities. An Apache application server and a MySQL database enable the persistent storage and allow the secure communication with the mobile device. The connection from the PhysioDroid app to the server is established in the* upload* package and data is transmitted both via the WIFI or the 3G network, as described in Section 5. The present prototype is deployed on the AdaBIO testbed, at the University of Granada. However, in a real setup, each health care provider, for example, insurance companies or public health organizations, would have their own server to which users' data would be gathered. Through authentication, only registered users can access the server, and depending on the access policies defined by the health care provider, authorization to access patients' health data is granted.

In case thousands of users made use of PhysioDroid, the server would run into scalability issues. This problem would get even worse if PhysioDroid became one of top Android Apps in Google Play and gets millions of installs. One of the main issues relates to the fact of having thousands of users accessing the server simultaneously. A second important matter refers to the storage of huge amounts of data, generated by a large number of users. Finally, the bigger the data sets are, the higher the computational processing costs are.

Cloud computing [40] and big data [41] are cutting-edge technologies devised to solve the aforementioned problems. PhysioDroid could exploit the benefits of cloud computing and big data to ensure scalability. For example, cloud providers offer load balancers, such as Amazon EC2 Elastic Load Balancing [42] or Rackspace Load Balancer [43], which would enable the distribution of PhysioDroid traffic across several servers. For massive data storage purposes, nonrelational cloud databases, such as Apache Cassandra [44], CouchDB [45], and MongoDB [46], are particularly suitable and also offered by most cloud providers. Moreover, frameworks like Apache Hadoop [47] provide cloud storage and large-scale processing functionalities, such as MapReduce [48], and could be of worth use in PhysioDroid.

We are working at the moment on a new version of PhysioDroid, which uses the* Open-Source, Web-Based, Framework for Integrating Applications with Social Media Services and Personal Cloudlets (OPENi)* (ICT FP7 Project OPENi, http://www.openi-ict.eu/) for the cloud storage of collected physiological data and their management.

OPENi offers the Cloudlet platform to store users data on the cloud. The OPENi Cloudlet platform provides application users, like PhysioDroid users, with a single location to store and control their personal data. The Cloudlet builds on the cloud database MongoDB and empowers application users to remain in control of their data. The control mechanisms are inherently secure and trustworthy, assuring the users data are not disclosed without their consent. Each PhysioDroid user will have their own Cloudlet to store their data. The Cloudlet functionalities will give the PhysioDroid users the maximum control of their data and over third party access to their data.

The PhysioDroid will use the Cloudlet API in order to upload the collected physiological data to the user's Cloudlet. The OPENi Encryption functionality, which is based on AES and one-time-keys, will allow on-the-fly encryption of the data collected by the PhysioDriod application preserving its confidentiality when uploading it from the mobile device to the user Cloudlet.

Not only will the PhysioDroid data be securely transmitted and stored into the user Cloudlet, but its secure and trustworthy access will be also granted by the OPENi Privacy and Security Framework. The OPENi permission visualization functionality will allow the PhysioDroid user to set up the data access rights via an intuitive graphical user interface, thus, defining who can access their data. These permissions and the authorization and authentication OPENi functionalities will ensure that the user physiological data is not disclosed to any malicious applications or users. Only the authorized parties, for example, the doctor, the clinician, or the family member, will be able to access the PhysioDroid data available in the users Cloudlet.

Last but not least, OPENi will allow the easy development of new applications that use the PhysioDroid data stored in the users Cloudlet and any combination of independent cloud-based services made available through the OPENi API Platform, as well as the development of processing and reasoning functionalities on the PhysioDroid data available on the Cloudlet.

## 7. On the Use of PhysioDroid

Two practical use cases for both average users and specialists in health and sports domains are described in the following.

One of the key features of the PhysioDroid system is the ability of presenting and detecting risk conditions. Some vital sign measures are easy to interpret, as they reach critical or extreme values (e.g., no respiration, too low/high skin temperature, or heart rate collapse). However, the interpretation of these very data in a more ordinary situation varies from patient to patient. For example, heart rate measurements differ depending on personal factors such as gender, age, weight, or height. The differences may be even higher for patients that suffer from illnesses or health conditions. Medical experts are capable of benefiting from all this information to correctly interpret the patient's vital signs. This medical knowledge could be easily incorporated by the specialist into the PhysioDroid system. The PhysioDroid app has been defined to allow specialists to customize the interval of interest (normality/abnormality) for each measured physiological sign, thus, adapting the visual representation and alarms to them. Taking advantage of this characteristic, a possible application could be referred to the persistent evaluation of the user's cardiac rhythm to detect abnormal situations and further trigger proactive policies. Once detected, and depending on the magnitude of the abnormality, the patient could be directed to follow specific guidelines provided by the doctor. Should it be required, an automatic emergency call/message may inform the closest clinical center.

In a clinical or emergency setting, the medical expert may also benefit from the use of the PhysioDroid system to accelerate the diagnostic. Patients often need to wait for some time before the device to run, for example, an ECG test or a respiration check, is available. The cost of these devices determines that they are normally shared among various specialists. The PhysioDroid system brings here a readily available alternative for a practical vital sign monitoring. Thus, for example, when the patient arrives to the clinical center the doctor may connect the PhysioDroid app to the wearable monitoring device to rapidly check the patient vital signs. Moreover, the monitoring may also be continuously performed in the ambulance on the way to the hospital or clinical center.

**Figure 6:**
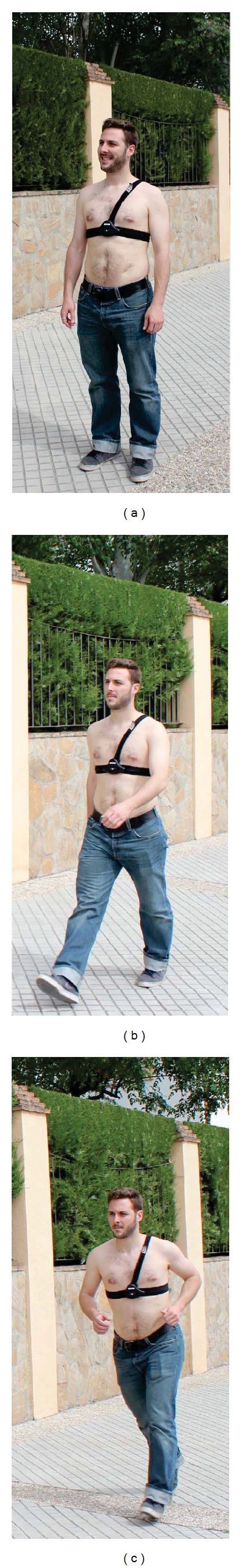
Examples of different intensity exercises monitored through the EQ01 sensors. User (a) standing still, (b) walking, and (c) running.

The PhysioDroid system is not just limited to the medical field but well-being, well-working, or other health-related domains. Here, an example for its use in sports is presented. Particularly, the idea is to assess whether the user reaches the target or training heart rate (THR) during the exercising. This has been shown to coincide with the phase when heart and lungs receive the most benefit from a workout. The approach basically consists of analyzing the heart rate while the user performs diverse intensity activities (e.g., Figure 6). HR ranges may be defined through diverse state-of-the-art methods extensively used in sports and fitness disciplines. From the literature, the Karvonen method [49], or more recently, the Zoladz method [50], offers an empirical way of defining diverse ranges regarding the intensity of the performed activities. The latter is particularly preferred, since it just requires to know the maximum heart rate (HRmax) to derive the exercise zones. The Zoladz method proposes five training heart rate zones or ranges (THR) that are respectively obtained as follows:

(1) $$\begin{array}{c} \text{THR}=\text{HRmax}-\text{Adjuster}\pm 5\;\text{bpm}, \end{array}$$

where zone 1 (recovery (aerobic)) → Adjuster = 50 bpm; zone 2 (endurance (aerobic)) →Adjuster = 40 bpm; zone 3 (stamina (aerobic)) → Adjuster = 30 bpm, zone 4 (economy (anaerobic)) → Adjuster = 20 bpm, and zone 5 (speed (anaerobic)) → Adjuster = 10 bpm. To obtain the HRmax several formulas based on the users' age and gender, information acquired by the PhysioDroid app during the user registration process, are available from the literature. Examples of these formulas are the one by Robergs and Landwehr (HRmax = 205.8 − (0.685 × age), [51]), Lund (HRmax (men) = 203.7/(1 + exp⁡⁡(0.033 × (age − 104.3))), [52]; HRmax (women) = 190.2/(1 + exp⁡⁡(0.0453 × (age − 107.5))), [53]) or Gulaty (HRmax (women) = 206 − (0.88 × age), [54]) among others. These formulas must be considered as average approximations, which perform well in most cases, but greatly depend on the user physiology and fitness. In fact, these models are of limited use on people with cardiac problems such as tachycardia, bradycardia, or arrhythmia [55], thereby this approach is rather planned for healthy people. In either case, all these formulas are automatically calculated and adapted to the user by the PhysioDroid system.

The previous use case is not only devised for the amateur user but for athletes and their coaches. Athletes may be continuously monitored during their exercises while the coach analyzes the measured information. This data could be of worth to improve training routines or avoid injuries normally caused from an inadequate warm up or cool down procedure.

## 8. Conclusions and Future Work

In this paper, a portable physiological and behavioral monitoring system devised for people health and wellbeing empowerment is presented. The system consists of a wearable monitoring sensor, a mobile device, and an optional remote persistent storage unit. The wearable monitoring device records diverse vital signs and transmits them to the mobile device on which a specifically developed Android app runs. The app acts as gateway supporting physiological data gathering and local storage, as well as data uploading to the remote storage for further processing. The application also allows the users to visually inspect the vital signs information collected through the wearable health sensor. This information is presented according to the users' level of expertise. Simple alerts and emergency calls are also features included as part of the app. Although the application has been here defined for the Equivital EQ01 monitor, it has been implemented in a way that little effort is required to make it compatible with any type of Bluetooth interfaced health monitor. Finally, a couple of use cases for the PhysioDroid system has also been suggested as part of this work.

Next steps aim at incorporating sophisticated data analysis and decision support techniques that may lead to a more profound description of the users' status and their evolution, key information for a customized, and personalized health care. As much as the number of users of the PhysioDroid system increases, more powerful resources should be also considered. The use of mainstream cloud solutions such as the ones provided by the OPENi platform is within the scope of current and future work. The authors also aim to shortly grant full and free access to the app through the Google Play store. Moreover, the source code will be also provided under GPL license so the app may be adapted to the particular requirements of the target problem.
